# Deficits in the Sensitivity to Pitch Sweeps by School-Aged Children Wearing Cochlear Implants

**DOI:** 10.3389/fnins.2016.00073

**Published:** 2016-03-03

**Authors:** Mickael L. D. Deroche, Aditya M. Kulkarni, Julie A. Christensen, Charles J. Limb, Monita Chatterjee

**Affiliations:** ^1^Centre for Research on Brain, Language and Music, McGill UniversityMontreal, QC, Canada; ^2^Auditory Prostheses and Perception Laboratory, Boys Town National Research HospitalOmaha, NE, USA; ^3^Department of Otolaryngology – Head and Neck Surgery, University of California San Francisco School of MedicineSan Francisco, CA, USA

**Keywords:** cochlear implants, children, pitch perception, speech prosody, intonation, emotion recognition

## Abstract

Sensitivity to static changes in pitch has been shown to be poorer in school-aged children wearing cochlear implants (CIs) than children with normal hearing (NH), but it is unclear whether this is also the case for dynamic changes in pitch. Yet, dynamically changing pitch has considerable ecological relevance in terms of natural speech, particularly aspects such as intonation, emotion, or lexical tone information. Twenty one children with NH and 23 children wearing a CI participated in this study, along with 18 NH adults and 6 CI adults for comparison. Listeners with CIs used their clinically assigned settings with envelope-based coding strategies. Percent correct was measured in one- or three-interval two-alternative forced choice tasks, for the direction or discrimination of harmonic complexes based on a linearly rising or falling fundamental frequency. Sweep rates were adjusted per subject, in a logarithmic scale, so as to cover the full extent of the psychometric function. Data for up- and down-sweeps were fitted separately, using a maximum-likelihood technique. Fits were similar for up- and down-sweeps in the discrimination task, but diverged in the direction task because psychometric functions for down-sweeps were very shallow. Hits and false alarms were then converted into d′ and beta values, from which a threshold was extracted at a d′ of 0.77. Thresholds were very consistent between the two tasks and considerably higher (worse) for CI listeners than for their NH peers. Thresholds were also higher for children than adults. Factors such as age at implantation, age at profound hearing loss, and duration of CI experience did not play any major role in this sensitivity. Thresholds of dynamic pitch sensitivity (in either task) also correlated with thresholds for static pitch sensitivity and with performance in tasks related to speech prosody.

## Introduction

Many children who were born deaf or lost their hearing very early in life are now routinely implanted with cochlear implants (CIs). These devices are remarkably useful in restoring speech intelligibility in quiet (Zeng, 2004). Most CIs currently available in the market use envelope-based coding strategies: they extract the within-channel temporal envelopes of speech to modulate trains of electrical pulses sent directly to the cochlea. Although the fine structure information is simply ignored in such strategies, envelope modulations are reproduced sufficiently well to enable reasonable intelligibility in quiet environments. The fine structure information (particularly that of low-order resolved harmonics) is, however, essential for accurate harmonic/complex pitch perception (e.g., Bernstein and Oxenham, 2005). Sensitivity to harmonic pitch is important not only for the perception of music (McDermott and Oxenham, 2008), but also plays a role in speech comprehension in terms of speech intonation (Cutler et al., 1997), vocal emotion (Murray and Arnott, 1993), lexical tone recognition (Kuo et al., 2008), or helping to separate a target speaker's voice from a competing background (Brokx and Nooteboom, 1982). As harmonic pitch is not coded or transmitted well to CI listeners, as a group they still face major challenges in their daily life with oral communication. Difficulties with recognizing the mood of a speaker, for instance, can have important consequences for a child's linguistic and social development (Schorr et al., 2009; Eisenberg et al., 2010; Geers et al., 2013).

Deroche et al. (2014) examined pitch sensitivity of 116 children, with either normal hearing (NH) or CIs, who were growing up in the US or in Taiwan. Using a standard procedure, a three-interval three-alternative forced-choice (3I3AFC) task with a constant stimuli presentation, they obtained the full psychometric functions for the fundamental frequency (F0) discrimination of broadband harmonic complexes at 100 and 200 Hz. They observed considerable deficits from CI recipients, which were independent of whether children spoke English or Mandarin (a tonal language), whether they were implanted at a very young age or later in life, and whether they had had an extensive or short experience with their CI. This lack of effects from plasticity-related factors radically contrasts with the large body of empirical evidence that early implantation is beneficial to many components of language development (Fryauf-Bertschy et al., 1997; Tyler et al., 1997; Nikolopoulos et al., 1999; Kirk et al., 2002; Lesinski-Schiedat et al., 2004; Svirsky et al., 2004; Dettman et al., 2007; Tomblin et al., 2007; Holt and Svirsky, 2008; Houston et al., 2012). One possible interpretation for this apparent discrepancy is that speech contains redundant information. A listener who does not receive much F0 information may be able to compensate for this missing cue by more strongly utilizing co-varying acoustic information within the signal, e.g., co-varying intensity or duration cues (Peng et al., 2009, 2012; Winn et al., 2012, 2013). The ability to use alternative acoustic cues or alternative listening strategies to successfully perform a given task may be facilitated by plasticity. In a simple psychophysical task with tightly controlled parameters such as that used by Deroche et al. (2014), there is no other way to do the task than by listening to the pitch. Without access to the fine structure, any listener would have to rely on envelope periodicity instead, which has a much poorer resolution than fine structure periodicity as seen from a summary autocorrelation function (e.g., Meddis and O'Mard, 1997).

To examine this early-implanted population further in a speech related task, where plasticity factors may potentially play a larger role, while placing a strong emphasis on the reception of F0 information, Chatterjee et al. (2015) measured voice emotion recognition by CI and NH children. Once again, CI children displayed considerable deficits in this task relative to their NH peers. Their results did not depend on age at implantation, providing no further support for the role of prior auditory experience even though this task used natural voice recordings as stimuli. However, NH children performed worse than NH adults when presented with noise-vocoded utterances; this finding was further corroborated by an effect of chronological age within NH children. This developmental effect in NH children with vocoded speech suggests that without prior experience, the auditory and language systems of the brain need to be more fully developed to process emotion in spectrally degraded speech. Similar findings have been reported by other investigators in the realm of speech recognition (Eisenberg et al., 2001; Nittrouer et al., 2009; Lowenstein et al., 2012).

F0 information is, however, not static in natural speech; instead, it continuously fluctuates over time. Performance in recognition of voice emotion, or in discriminating between a question and a statement, must strongly depend upon the dynamic tracking of F0 information over time. This argument is even more relevant to tonal languages where children are continuously exposed to rapid pitch inflections within syllables and must learn to categorize them into separate tones early in life (Chao, 1968; Howie, 1976; Liu and Pell, 2012). Dynamic pitch sensitivity may therefore represent a finer and more informative estimate of a listener's ability to use voice pitch information in real life. In addition, Deroche et al. (2014) had found significant effects of chronological age in the static F0 discrimination task, which here incited us to compare children and adults. CI adults can have a very different profile than CI children: four in this study were post-lingually deaf while all children were pre-lingually deaf (i.e., they learnt to communicate without normal acoustic input). Consequently, adult CI data offer an additional avenue for the examination of the role of prior auditory experience. To summarize, the present study examined two main hypotheses: (1) do CI listeners display deficits in their sensitivity to dynamic pitch compared to the NH peers, and (2) are these expected deficits smaller or larger in the pediatric or in the adult populations? Sensitivity to linear sweeps of F0 was thus measured in a 1I2AFC sweep-direction-labeling task (up or down) and a 3I2AFC sweep-direction-discrimination task (which of two sweeps was in a different direction relative to a reference sweep). The choice of having two tasks simply served to examine the possibility that CI listeners could somehow attend to uncontrolled differences between successive sounds without necessarily having a sense of pitch direction. This possibility was tested by correlating the sweep sensitivity thresholds, not only between the two tasks, but also with static thresholds. Finally, a third goal was to assess the extent to which these pitch sensitivity measures could inform us about the use of voice pitch information in a child's social and linguistic development. To this aim, all thresholds (static or dynamic) were correlated against the same children's performance in the emotion recognition task (data published by Chatterjee et al., 2015), in order to find out which of these different measures held any predictive power for the reception of speech prosody in real life.

## General methods

### Listeners

Four groups of listeners participated. There were 21 NH children, 23 CI children, 18 NH adults, and 6 CI adults. The chronological age of the NH children varied from 6.1 to 18.1 years; that of the CI children varied from 8.1 to 17.9 years. Age at implantation of the CI children varied from 1 to 12 years. Fifteen of them were profoundly deaf from birth while eight others had profound hearing loss beginning between 3 months and 3.5 years of age. Their duration of CI experience varied from 4.9 to 16.7 years. Six of the CI children were unilaterally implanted, while 17 were implanted on both sides. Four CI adults were unilaterally implanted and two implanted on both sides. The listeners implanted bilaterally were always tested on the side implanted first. A few subjects had sufficient residual acoustic hearing to wear a hearing aid on the contralateral ear. However, when there was any chance that the subject could hear from the contralateral ear, because of a second implant, a hearing aid, or some residual hearing, the CI processor or hearing aid was removed and the contralateral ear was plugged with ear-foam. Table 1 provides further details on the demographics of children and adults.

**Table 1:** **Demographics of the four groups of listeners**.

	**Chronological age mean (std.) [min–max]**	**Age at implantation mean (std.) [min–max]**	**Duration of CI experience mean (std.) [min–max]**	**Age at profound hearing loss mean (std.) [min–max]**
NH adults	34.8 (11.4) [21.2–50.9]			
NH children	11.8 (3.4) [6.1–18.1]			
CI adults	57.2 (3.6) [52.5–61.2]	49.3 (7.3) [41.0–59.0]	7.9 (4.9) [2.2–13.0]	20.1 (18.5) [1.5–49.0]
CI children	13.0 (3.0) [8.1–17.9]	2.6 (2.3) [1.1–11.9]	10.4 (3.5) [4.9–16.6]	0.5 (1.1) [0.0–3.5]

Among the CI children, 13 had a *Cochlear* device: 6 wore a Nucleus 24, 3 wore a Nucleus Freedom, 2 wore a Nucleus N6, 1 wore a Nucleus 5, and 1 wore a Nucleus CI512, all using the ACE speech processing strategy. Ten others had an *Advanced Bionics* device: 5 had a Clarion CII, 3 wore a Clarion HiRes90k, and 2 wore a Naida, with different processing strategies (HiRes, SAS). Among the CI adults, five had a *Cochlear* device: 1 wore a Nucleus 24, 2 wore a Nucleus Freedom, 1 wore a Nucleus N6, and 1 wore a Nucleus CI512, using the ACE strategy. The remaining CI adult had an *Advanced Bionics* device (HiRes 90k). Stimulation strategies were therefore all envelope-based. All implanted listeners used their clinically assigned settings. All participants were tested in both tasks (the sweep direction discrimination task and the sweep direction labeling task).

### Stimuli

F0-sweep stimuli were generated from harmonic complexes with n partials, all in sine phase having equal amplitude. Note that the amplitude of different partials varies considerably for a voice or a musical instrument, but these variations may be a potential confound because listeners could entirely focus on amplitude changes around a specific resonance as fundamental frequency varies. Therefore, it was preferable here to equalize all partials in amplitude to compel listeners to integrate information across all the spectral channels available to them. The value of n was chosen as the Nyquist frequency divided by the highest F0 reached during a given sweep. To eliminate cues based on the spectral edge pitch at high frequencies, stimuli were low-pass filtered at 10 kHz using Butterworth sixth-order filter with a slope of -30 dB per octave. Stimuli were 300-ms long in order to stay close to the syllabic rate, with 30-ms onset and offset ramps. The inter-stimulus interval (when applicable) was also 300 ms. The F0 of the complex varied linearly with logarithmically-spaced rates: 0.5, 1, 2, 4, 8, 16, 32, 64, and 128 semitones per second. Occasionally, extremely shallow sweeps at 0.25 semitones/sec and extremely steep sweeps at 256 semitones/sec were presented when a given listener seemed to perform particularly well or particularly poorly. The base F0, defined as the starting F0 for up-sweeps and the ending F0 for down-sweeps, was chosen randomly from one trial to the next within a rectangular distribution between 100 and 150 Hz. An important experimental choice was that sweeps with opposite direction always shared the same F0 range. For instance, a rising tone, starting at 120 Hz with a rate of 4 semitone/s (i.e., reaching a F0 of 128.6 Hz after 300 ms) was always presented against a falling tone starting a 128.6 Hz with a rate of -4 semitone/s. Note that this was still true in the single-interval labeling task even though the two sweeps (up and down covering the exact same F0 range) could be separated by many trials due to the random presentation of each condition. This choice as well as the decision to rove the base F0 were aimed at discouraging listeners from completing the tasks based on the average of F0 values, or the highest or lowest F0 values, covered during the sweeps. All stimuli were equalized at 65 dB SPL but presented with ±3 dB level roving. Note that this level roving was in principle not necessary: since up- and down-sweeps covered the exact same range of F0, the loudness of both sweeps should be equal. The loudness of steep sweeps may well differ from that of shallow sweeps, but this would not help in choosing between up and down. Thus, the level roving was used here simply as a way to discourage any loudness-based strategies.

### Protocol

The protocol of the study was first explained carefully to subjects. There was no need for a sign language interpreter because all implanted subjects had sufficiently good speech understanding. After obtaining informed consent from both children and parents (or adults alone), the listeners were invited to seat in the auditory booth and started the practice blocks. The first task followed a 1-interval, 2-alternative forced choice (1I-2AFC) procedure: a single sweep was presented, either rising or falling, and the listener was asked to report whether the pitch was going *up* or *down*. The second task followed a 3-interval, 2-alternative forced choice (3I-2AFC) procedure: one reference sweep was presented, either rising or falling, followed by two sweeps, one identical and one in opposite direction, all at the same rate, and the listener was asked to report which interval sounded *different* from the reference. The target was placed with equal probability in the second or third interval. Each subject performed the direction task first and the discrimination task second. This fixed testing order was decided after a few subjects reported, during preliminary testing, that the discrimination task was cognitively more demanding. Even though this choice necessarily opens the possibility that performance could be lowered in the discrimination task due to fatigue effects, for children as young as 6 years old, it seemed simply preferable to start with the easiest task and not lose any motivation. Each task was preceded by one or several practice blocks.

Practice blocks differed from test blocks in two respects: (a) stimuli were presented without level roving (but always with roving of base F0); and (b) there were only 20 trials (10 ups and 10 downs, at random) measuring performance at 128 semitones/sec. The criterion for starting a test block was to achieve a performance of 80% or more, averaged over the two directions. This was typically achieved at the first block for NH listeners but over two or three more blocks for CI subjects. Occasionally, a few subjects (two NH children, one CI adult and one CI child) would not manage to pass the 80% criterion at 128 semitones/sec, in which case a new practice block was run on sweeps of 256 semitones/sec. If performance was still below 80%, the task was abandoned as performance would presumably not have improved with steeper rates. The percept of a purely temporal pitch only exists within a relatively narrow range: a broadband noise modulated at 400 Hz and beyond is simply perceived as stationary, both for NH listeners (Burns and Viemeister, 1976) and CI listeners (Zeng, 2002). With extremely steep rates, such as 256 semitones/sec, the stimuli swept over as much as 6.4 octaves over their 300-ms duration, meaning that half of such stimuli would presumably no longer lie within the existence region of temporal pitch. Thus, there was no point in examining sweeps steeper than 256 semitones/sec, at least for CI listeners.

A test block consisted most often of 140 trials (7 sweep rates by 2 directions, tested 10 times each), presented in random order. For NH listeners, these rates were typically 0.5, 1, 2, 4, 8, 16, and 32 semitones/sec, while for CI listeners, they were typically 2, 4, 8, 16, 32, 64, 128 semitones/sec. Before running a second test, the experimenter had a chance to look at the performance obtained in the first block. When it appeared that the scale was inadequate, either because performance was at floor or at ceiling, the experimenter could change the rates for subsequent blocks. For example, some NH adults achieved 100% performance in both up- and down-sweeps at rates as low as 4 semitones/sec. For those, a second test could focus on rates of 0.25–8 semitones/sec in a block of 120 trials, or simply 0.5–8 semitones in a block of 100 trials. Similarly, some CI subjects achieved near-chance performance for all rates, while having passed the 80% criterion during practice. For those, a second test could focus on rates between 32 and 256 semitones/sec in a block of 80 trials. When time allowed (and depending on the children's willingness to participate) a third, fourth, and fifth test block could be run, on the direction task, the discrimination task, or both.

The interface was similar to that described in Deroche et al. (2012): a cartoon became animated over the auditory presentation of each interval in synchronization with the visual presentation of a button. For the direction task, after the single interval presentation, button two and three appeared on the screen with the labels “up” and “down,” and the listeners provided their response. For the discrimination task, at the end of the third interval, the three buttons and three cartoons reappeared on the screen and the listeners clicked on the button for which the sound was perceived to be different. In order to gauge the assiduous or dabbler behavior of a given participant in each task, response time (RT) was recorded for each trial, from the end of the last stimulus presentation to the instant the subject pressed a response key/button. However, subjects were not aware of it; they were instead instructed to be as accurate as possible in a timely manner. Feedback was provided via smiley faces (happy, excited, sad, or disappointed) and by winning or losing points. The experimenter provided verbal support and encouragement to boost the child's motivation and attention. A typical experimental session lasted 45–75 min. Short breaks were offered in between each test block. All listeners were paid for their participation. Protocols were approved by the Institutional Review Boards of the two institutions at which this study took place.

### Equipment and testing sites

The study took place at two research facilities. Data for 33 subjects (12 NH adults, 6 NH children, and 15 CI children) were collected at the Music Perception Laboratory of Johns Hopkins Hospital in Baltimore. Data for 35 subjects (6 NH adults, 15 NH children, 6 CI adults, and 8 CI children) were collected at the Auditory Prostheses and Perception Laboratory of Boys Town National Research Hospital (BTNRH) in Omaha. Experimental setups were largely similar. Signals were always sampled at 44.1 kHz and a 16-bit resolution, presented via an external soundcard (Edirol UA) and a single loudspeaker, located ~2 feet from the subject, at an average level of 65 dB SPL. Loudspeakers (Sony SS-MB150H at Johns Hopkins, and Grason Stadler GSI at BTNRH) were placed directly facing the subject, and the user-interface was displayed on a monitor located inside the booth. Listeners provided their responses using a touch-screen, a keyboard, a joystick, or a mouse, depending on the available equipment and the child's preference. Note that there were no significant differences between data (within a given population) collected at the two sites.

## Data analysis

First, performance was averaged over all test blocks, i.e., between 10 and 50 trials per rate and per direction. Second, since both tasks followed a 2AFC procedure, hit rates were taken as the performance of up-sweeps and false alarm rates were derived from 1.0 minus the performance of down-sweeps. Hits and false alarms were then converted into d′ and beta data (Green, 1960). In order to compare sensitivity across subjects and across tasks in a fair manner, thresholds had to be extracted at a chosen value of d′. This was achieved by fitting the performance data using the maximum-likelihood technique described by Wichmann and Hill (2001a,b). This technique is particularly useful here as it gives more weight to those data points that have been measured over a larger number of trials. It was consequently very well suited to the present data that were collected over several test blocks on possibly different ranges of sweep rate. The underlying psychometric function was modeled using a Weibull function, and varied over a scale which was log-transformed using the formula 3 + log_2_(rate). A priori distributions for the Weibull parameters served to guide the fitting procedure. The lower bound was not fixed at chance level but instead had a Gaussian prior with a mean of 50% and a standard deviation of 10%. The upper bound had a Gaussian prior with a mean of 0% and a standard deviation of 30% to allow for inattention mistakes at rates that should have been trivial. The parameter related to the inflection point had a Gaussian prior with a mean of 5 (i.e., a sweep rate of 4 semitones/sec) and a standard deviation of 5. The parameter related to the slope had a Gaussian prior with a mean of 10 and a standard deviation of 10. Note that these distributions were chosen to be sufficiently broad to encompass all the different populations and tasks. Once fits were obtained for the performance of up-sweeps and down-sweeps separately, d′ and beta values were recalculated from these Weibull fits on a very finely grained logarithmic scale. Thresholds could then be extracted at any chosen value of d′. A d′ of 0.77 was chosen to ease comparisons with previous studies.

## Results

### Population data

**Figure 1:**
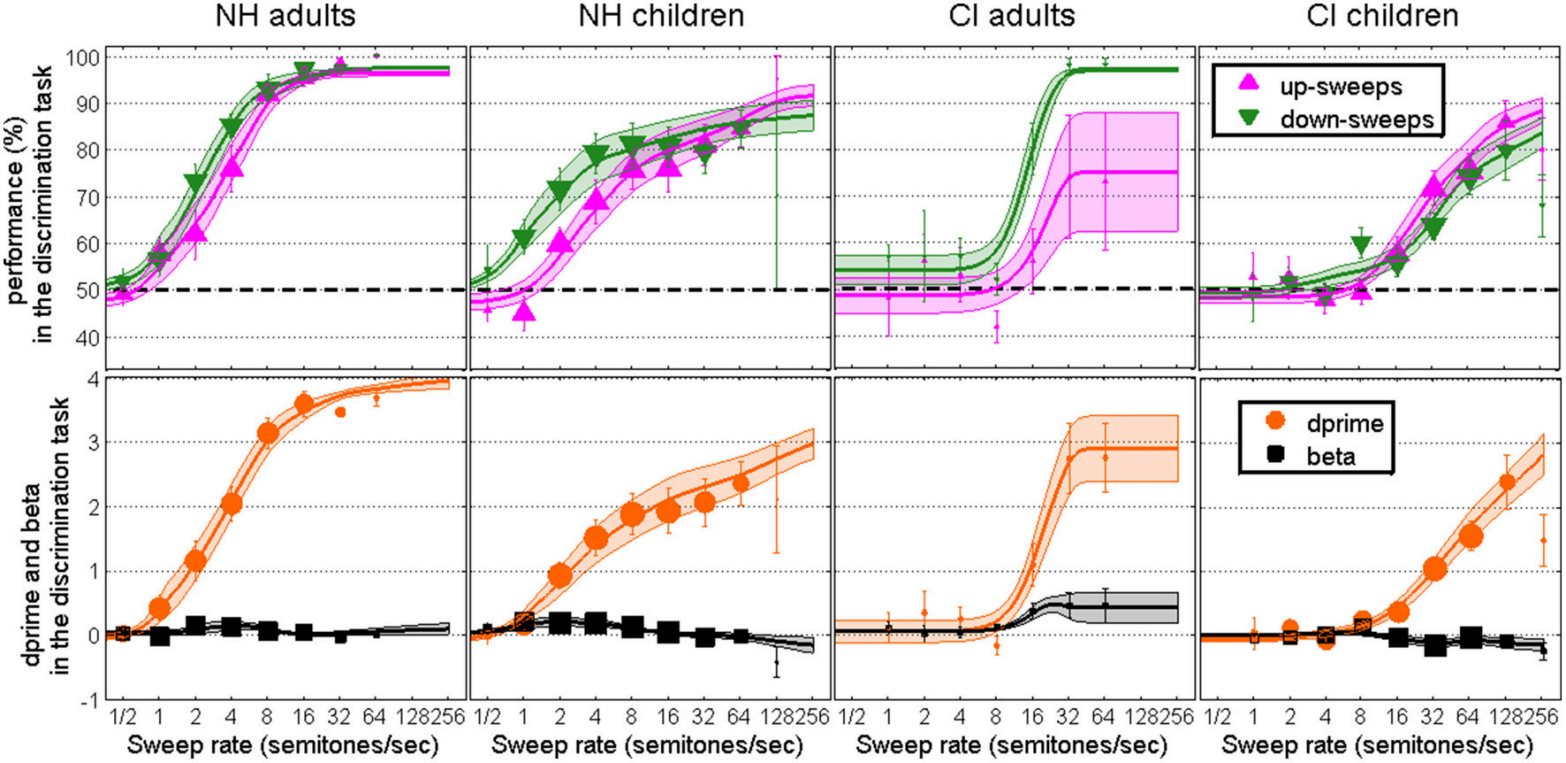
**Performance data (top), converted into d′ and beta data (bottom), in the 3I-2AFC task of F0 sweep discrimination as a function of rate, for NH adults, NH children, CI adults, and CI children**. Symbols represent the data collected, while lines and surfaces represent the Weibull fits.

**Figure 2:**
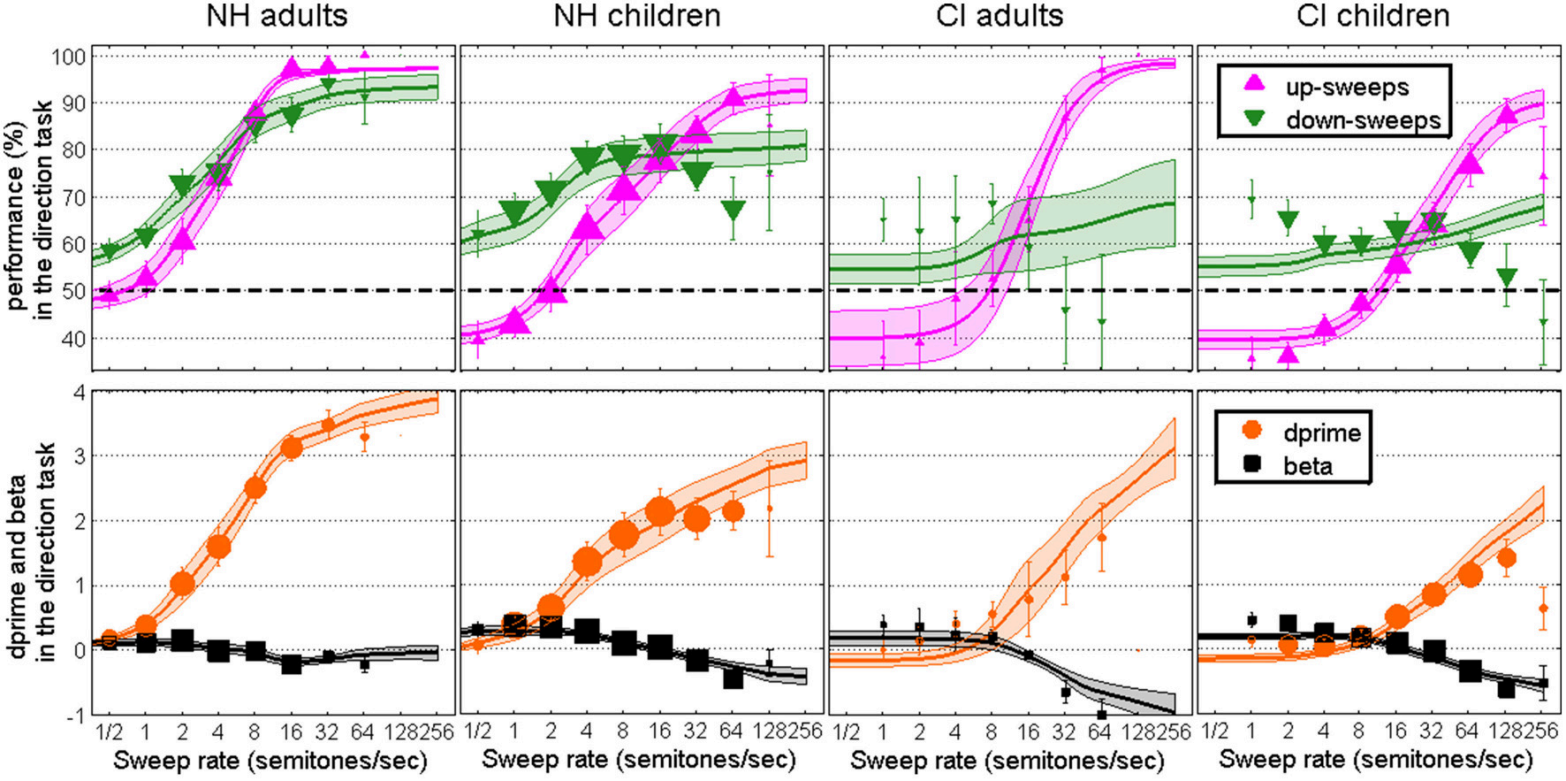
**Same as Figure 1 in the 1I-2AFC task of F0 sweep direction**.

**Figure 3:**
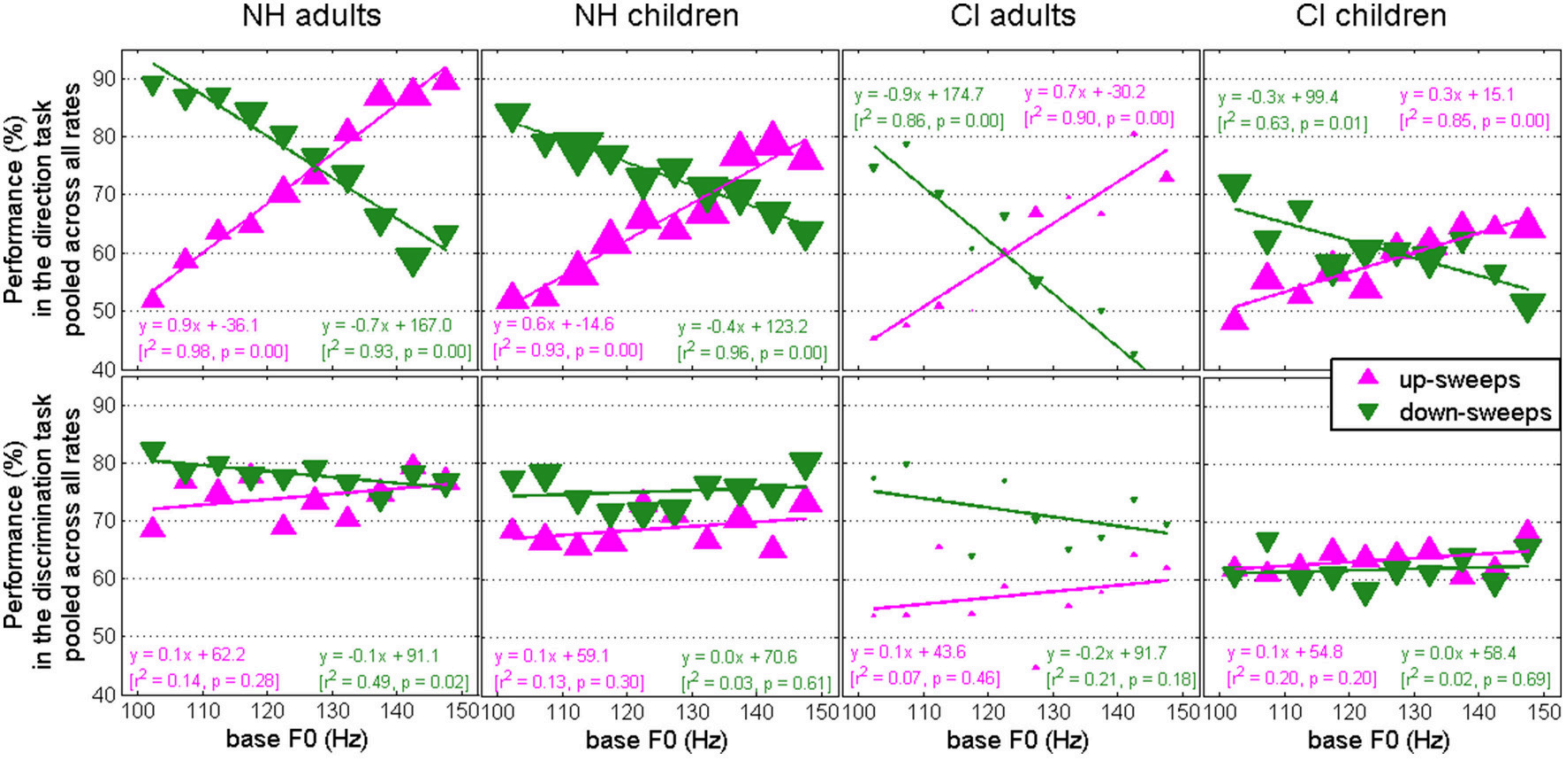
**Performance pooled across all rates and all subjects within a population, as a function of base F0, from which sweeps were constructed, which was roved between 100 and 150 Hz**. There was a large influence of base F0 for the direction task **(top)**, but none in the discrimination task **(bottom)**.

**Figure 4:**
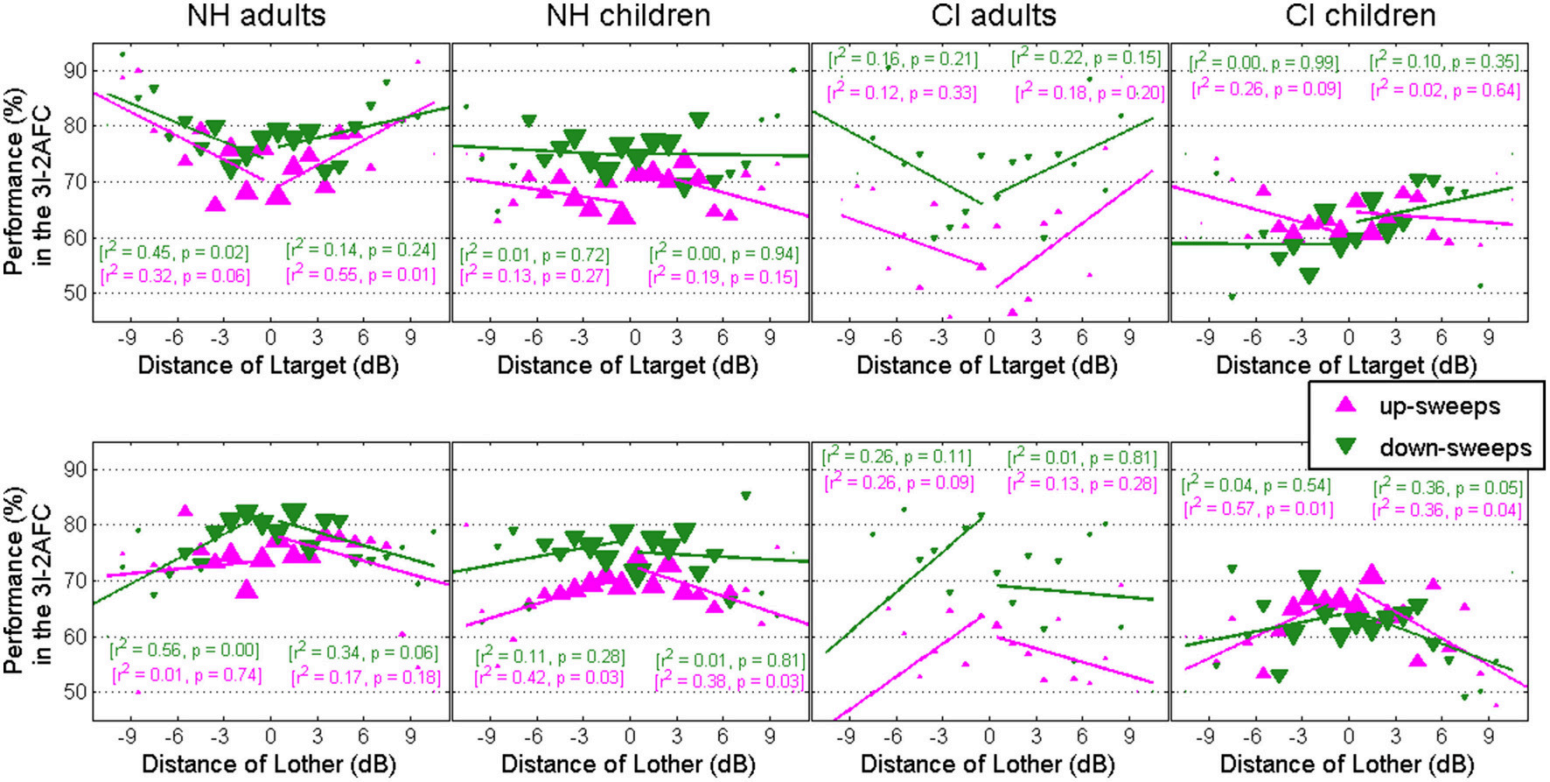
**Performance for the discrimination task only, pooled across all rates and all subjects within a population, as a function of the difference between the target level and that of the two other intervals (top) or the difference between the non-target level and that of the two other intervals (bottom)**.

**Figure 5:**
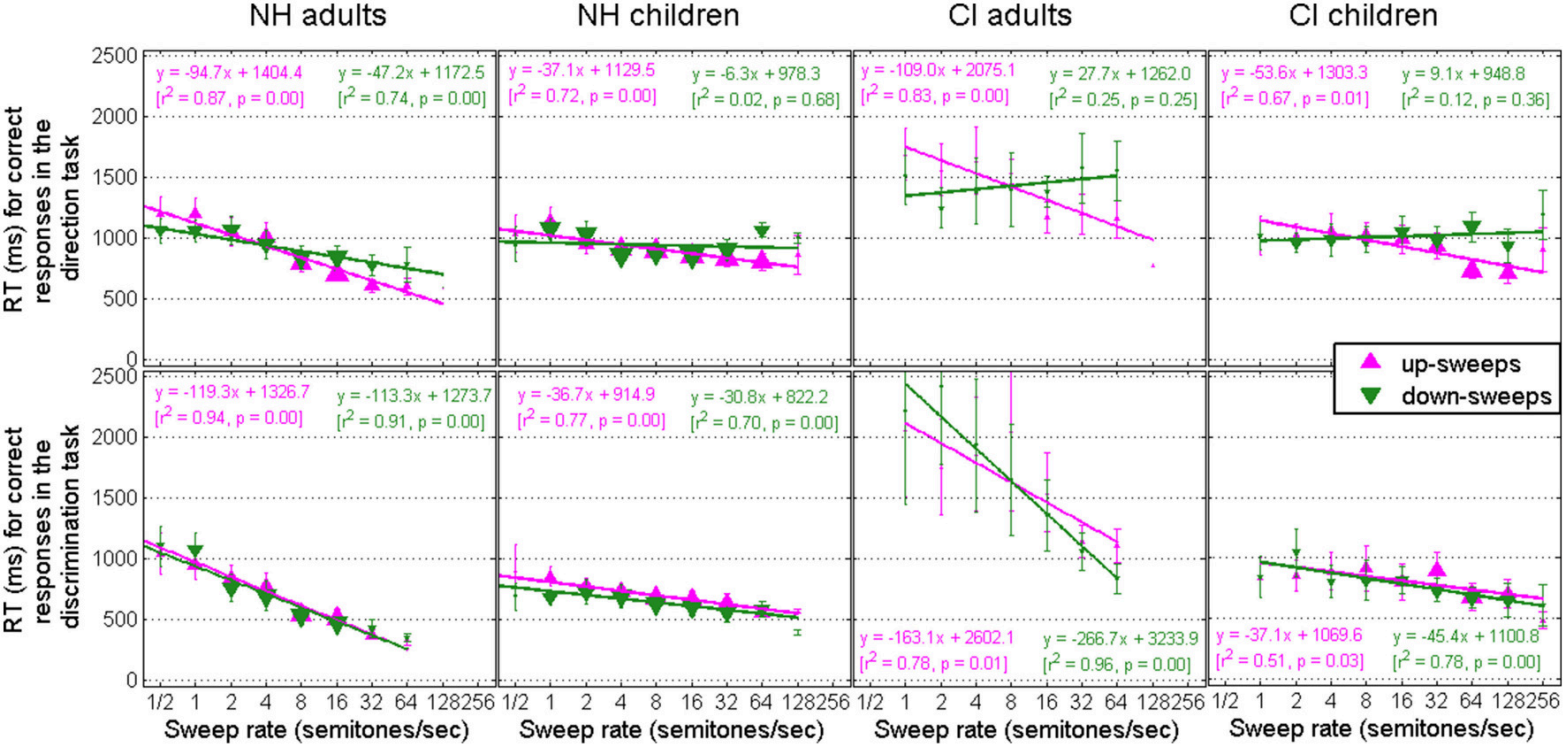
**Response Time data in the direction (top) and discrimination (bottom) tasks for trials that provided correct responses**. The consistent decrease in RT with rate is evidence of good behavior.

All the 18 NH adults were able to perform both tasks with relative ease. In contrast, two NH children, one CI adult, and one CI child, could not pass the criterion of 80% correct performance during practice and were excluded from further data collection. In addition, data for the discrimination task could not be obtained for four NH children and one CI child, due to a lack of time. Of the remaining subjects, data obtained in some cases (two NH children and three CI children in the direction task; two CI children in the discrimination task) did not yield a measurable threshold at d′ = 0.77 because performance remained near chance for all rates, but such data still contributed to the population data represented in Figures 1–**5**.

The top panels of Figure 1 show the performance data and the bottom panels show the two parameters of signal detection theory obtained in the *discrimination task*, across the four populations. Here and later, symbols represent the weighted mean of the data and error bars indicate one weighted standard error of the mean. Weights reflect the total number of trials run across subjects, and are proportional to the size of the symbols. For example, it can be seen on the left panels that NH adults were more rarely tested at 32 and 64 semitones/sec than at the six rates below. The size of symbols is also consistent across populations, which is why data from the CI adults (the smallest group) are represented by smaller symbols than any other population. It can also be seen that NH children (middle left) were very rarely tested at 128 semitones/sec, compared to CI children (right panels). The use of such weights ensured that sweep rates tested more reliably within a subject, and more consistently across subjects, would count more toward the mean and standard error than rates tested very occasionally. Note that at the very occasional sweep rate of 0.25 semitones/sec (not represented in the figures) performance was at chance for all subjects. At least for durations of 300 ms, stimuli with sweeps shallower than 0.5 semitones/sec sound essentially static. There is no point in testing them. Lines and shaded areas represent the weighted means and weighted standard errors of the Weibull fits. Here, weights reflected the total number of trials run for a given subject, meaning that the fit for one subject could have more influence on the average fit than the fit of another subject who had been tested less extensively.

Overall, the discrimination task provided orderly data. Performance was close to 50% correct at very low rates, showing no systematic bias toward up or down responses in any of the subject groups. At high rates, there was not much bias either (except perhaps for CI adults) such that beta remained fairly flat across all rates. For NH adults, d′ increased very steeply beyond 2 semitones/sec. For NH children, d′ increased at low rates as well, but asymptoted at about a value of 2.0, which was presumably caused by some inattention errors and the inclusion of subjects who provided chance-level data across all rates, reducing maximum performance to 80–90% correct. For CI subjects on the other hand, d′ was clearly at floor up to about 8 semitones/sec and increased slowly beyond, more slowly for children than for adults.

Goodness-of-fit was evaluated using the method advocated by Wichmann and Hill (2001a,b). All fits (ups and downs, for all four groups) were within the 95% confidence limits of the distribution of the Monte-Carlo-generated correlation coefficients between the deviance residuals and the percent correct predicted scores. This confirms that the Weibull function was an appropriate choice of underlying shape for the present data. However, several fits (4 out of 36 fits for NH adults, 10 out of 30 fits for NH children, 3 out of 10 fits for CI adults, and 10 out of 44 fits for CI children) were beyond the 95% confidence limits of the Monte-Carlo-generated deviance distributions. This lack of fit was partly caused by “noisiness” at shallow rates. With a 2AFC task, it often happens that subjects get lucky or unlucky in a very challenging condition, resulting in positive and negative deviances across the lower asymptote of the fits. This lack of fit was perhaps exacerbated in the pediatric populations where inattention mistakes were not homogeneous and resulted in additional deviances toward the upper asymptote of the fits.

Figure 2 shows similar plots as Figure 1 but for the *direction task*. Data for this task were less orderly than those in the discrimination task because (1) performance did not fit nicely at chance level at very low rates: there was a systematic bias in which listeners responded “down” more often and therefore obtained overall better performance for down- than for up-sweeps; and (2) there was a reverse problem at high rates whereby NH children, CI adults and CI children were very reluctant to respond “down.” In other words, no matter whether the sweep was going up or down, listeners were more likely to respond “up” to steep sweeps. Apart from these two anomalies, results were similar across the two tasks. NH adults had a very steep increase in d′ beyond 2 semitones/sec. NH children had a slower increase in d′ with sweep rate and asymptoted at d′s of about 2.0. For the CI populations, d′ did not increase until the sweep rate was beyond 8 semitones/sec and could not exceed about 1.5 even for the steepest sweeps. All fits were within the 95% confidence limits of the distribution of the correlation coefficients between the deviance residuals and the percent correct predicted scores, but several fits (2 out of 36 fits for NH adults, 11 out of 38 fits for NH children, 4 out of 10 fits for CI adults, and 9 out of 42 fits for CI children) were beyond the 95% confidence limits of the deviance distributions. More specifically, for down-sweeps, the anomalies aforementioned resulted, for some subjects, in a decline of the psychometric function at steep rates. The psychometric functions, necessarily monotonic, could not follow such decreases in performance. This is clearly visible in Figure 2 for CI adults and CI children where the “best” fits had to be extremely shallow. This is visible to a smaller extent for NH children at a rate of 64 semitones/sec. Since the fits for down-sweeps were mostly flat in those cases, the d′ fits were largely based on the fits for up-sweeps.

### Anomalies of the direction task

The two anomalies observed in the direction task were unexpected, and it is reasonable to ask what caused such biases and whether they were related one another. As will be shown in Section F0 Roving, listeners did in fact try to use alternative strategies than the sweep direction (despite the experimenter's instructions), specifically in the direction task. When listeners were presented with shallow sweeps, they simply responded “up” when the sweep covered a relatively high F0 range, e.g., between 125 and 150 Hz, and responded “down” when the sweep covered a relatively low F0 range, e.g., between 100 and 125 Hz, regardless of the sweep direction. This would seem a fairly logical strategy if, indeed, listeners heard a static pitch. However, since base F0 was roved, this strategy was not successful and the probability of a correct response remained unchanged at 50%. Therefore, such a strategy should have been of no consequence for performance, and importantly, it should not have favored the performance of one direction over the other. However, the scenario was a little different when sweeps went beyond the F0 roving range of 100–150 Hz. In comparison with many shallow sweeps that remained in the 100–150 Hz range, steep sweeps reached a higher F0 range either from the start (for down-sweeps) or by the end (for up-sweeps) of the stimuli. A strategy based on long-term average of F0s would always choose “up” as soon as the median F0 is raised above the roving range. This is most likely the strategy that several subjects followed, disregarding the direction of very steep sweeps to rely exclusively on long-term average of F0. Furthermore, it follows that these same listeners could have started to respond “down” to most sweeps whose median F0 was within the 100–150 Hz range, by contrast with the steep sweeps whose median went beyond, resulting in the opposite bias in favor of down-sweeps at shallow rates. Therefore, these two anomalies are akin to two sides of the same coin. They did not occur in the discrimination task, presumably because the F0 range is explicitly identical across the three intervals, so that listeners would have to discard any internal reference of pitch range constructed over trials.

### F0 roving

The base F0 (defined as the starting F0 of up-sweeps and the ending F0 of down-sweeps) was roved within a rectangular distribution between 100 and 150 Hz for each trial. It was of interest to examine the influence of this roving on performance, between the two tasks. To this end, data were pooled across all rates and across all subjects of a population, and divided into 10 bins of base F0, from 100 to 150 Hz in 5-Hz steps. For each bin, average performance was calculated for up- and down-sweeps, and represented in Figure 3. The size of symbols is proportional to the number of trials within a bin, but since the F0 roving distribution was rectangular, all bins ended up with similar number of trials, for a given population. A weighted correlation was performed for each population and each task, and the regression lines, *r*^2^ and *p* values are shown in each panel.

In the direction task (top panels), there was a significant correlation between performance and base F0 for each population and direction, with *r*^2^ between 0.63 and 0.98, reflecting that this task was strongly influenced by the base F0. Note that all these correlations would survive Bonferroni corrections for multiple (eight) correlations. The two adult populations obtained steep regression lines, with positive and negative signs for up- and down-sweeps, consistent with the idea that listeners used the F0 range as an additional cue (although it was not informative in the task). The slopes of 0.7–0.9 in the adult data imply a 35–45% change in performance within the 50-Hz range of base F0. Both pediatric groups obtained slightly shallower slopes, 0.3–0.6, implying a change of 15–30% within the 50–Hz range.

In the discrimination task (bottom panels), only one case showed a significant correlation with base F0, which does not survive Bonferroni corrections, and slopes were about ±0.1. To put it otherwise, base F0 never influenced performance by more than 5% within the 50-Hz range. Thus, base F0 played little role in the discrimination task, depicting a clear contrast with the direction task.

This analysis was also performed on an individual rate basis. Correlations between performance and base F0 were strong for low rates up to about 8 semitones/sec but these correlations declined at higher rates. This pattern was homogeneous across the four populations. This would suggest that base F0 largely influenced the responses when sweeps were relatively shallow but lost its influence when sweeps became steep. This is due to the fact that the 100–150 Hz range of base F0 became progressively a single category of relatively low pitches put in opposition with the high pitch range elicited by steep sweeps. As an example, a sweep at 128 semitones/sec would have reached 1011 or 1286 Hz whether it was based on 110 or 140 Hz, and both cases became one single case in which a large portion of the sweep ranged in a much higher pitch range than many other, shallower, sweeps. Therefore, the alternative strategy based on long-term average F0 was presumably employed across all rates but only translated to strong correlations with base F0 at shallow rates.

### Level roving

The presentation level of each interval was roved within a rectangular distribution between 62 and 68 dB SPL. This rove applied to the acoustic input would translate to different degrees of rove of electrical current for implanted listeners, depending on their settings. We were curious to examine how much of an influence on performance could this level roving have had, and whether this affected implanted listeners more particularly. To this end, data were pooled across all rates and across all subjects of a population, and divided into 12 bins of level roving, from −3 to +3 dB in 0.5-dB steps. First, we considered the level of the target interval alone. In both tasks, there was no correlation between performance and the level of the target interval for any direction and any population (not represented). Put it simply, it did not matter that stimuli were presented at 62, 65, or 68 dB SPL. Second, we considered the level of the target interval relative to that of the other two intervals in the discrimination task. After all, the target level may not matter on its own; if both the reference and the non-target interval were presented close to 62 dB SPL, a target presented at 63 dB SPL might stand out as louder. To this aim, we derived a simple estimate of distance between the level of the target relative to those of the two other intervals, by using (L_target_ − L_reference_) + (L_target_ − L_other_). Similarly, we derived an estimate of distance between the level of the non-target interval relative to those of the two other intervals, by using (L_other_ − L_reference_) + (L_other_− L_target_). Data were pooled across all rates, and divided into 24 bins of distance, from −12 to +12 dB in 1-dB steps. The distribution of these distance estimates is no longer rectangular because there were many more trials in which the target was located in between the two other intervals, i.e., a distance close to 0 dB, than trials in which the target was located on the opposite extremity of the scale relative to the two other intervals. Figure 4 shows performance in the discrimination task as a function of target distance (top panels) and non-target distance (bottom panels). In the top-left panel, it can be seen that when the target was substantially softer or when it was substantially more intense than the two other intervals, NH adults were more prone to choose the target interval, resulting in a better performance in both cases. Thus, performance for both up- and down-sweeps followed a V-shape as a function of target-distance. Conversely, in the bottom panels, performance followed an inverse V-shape as a function of distance of the non-target interval. Listeners were more prone to pick the wrong interval when it stood out from the two other intervals, either because it was softer or because it was more intense. Even though these trends may follow some curvature, the linear trends illustrate the general shape of these effects, but note that none of these correlations would survive Bonferroni corrections considering 16 correlations. A similar pattern occurred for CI adults, even though none of these correlations revealed to be significant. For the two pediatric populations, there was relatively little influence of target distance and a non-negligible influence of non-target distance, particularly for up-sweeps. These loudness-induced changes in performance were contained within 10–20%. As a conclusion, when the task is simply about picking the odd interval, listeners were to some extent influenced by loudness differences provided that they were sufficiently salient, i.e., distances beyond about ±6 dB, which were helpful when they happened to cue the target interval, and harmful when they happened to cue the non-target interval. There was, however, no apparent indication that these loudness cues affected CI listeners more than they affected NH listeners. If anything, they seemed to affect adults more than children, irrespective of the hearing status.

### RT data

For each trial, RT was recorded and stored depending on whether the response provided was correct or incorrect. Figure 5 shows RT data for correct responses in the direction task (top panels) and in the discrimination task (bottom panels), as a function of sweep rate. Once again, the size of symbols is proportional to the number of trials (correct or incorrect, separately) collected at a given rate, which served as weights in the linear regressions performed in each panel. The correlation between RT and sweep rate was significant in all panels of Figure 5, except for three cases that occurred for NH children, CI adults, and CI children in the direction task specifically for down-sweeps. Those were the same three cases where the anomalies (discussed in Section Anomalies of the Direction Task) were observed. This shows how sensitive the RT measure is to the performance data. Presumably, in those situations, listeners were conflicted between two strategies: relying on the sweep direction or relying on the long-term average F0, which led them to take longer to respond and also make mistakes. Apart from those three cases, the general pattern of RT data is evidence for a generally good behavior (Luce, 1986). When listeners found the task easy to perform, they were more efficient at doing it. When they were not sure about the answer, they took longer to make the right choice. To delve into details, one can examine the slope of these correlations. For every doubling of the sweep rate, NH adults took about 100 ms less to provide correct answers. NH children took about 30 ms less, CI adults between 30 and 260 ms less, and CI children about 50 ms less to provide correct answers. It is particularly interesting to observe that RT continued to decrease at high rates even though d′ could have asymptoted. For instance, d′ largely asymptoted beyond 8 semitones/sec for NH adults and NH children in both tasks, but RT continued to decrease by an appreciable amount. This sort of phenomenon is sometimes referred to as “listening effort.” A given task may be less demanding to listeners than another, and is sometimes not revealed by any difference in performance if performance is already close to ceiling, but may be revealed by a simple measure of RT. Consistent with this good behavior, mistakes also led to longer RT than correct responses (Figure not shown). Mean RT for mistakes was relatively stable as a function of rate, and was as long as the mean RT for correct responses in the hardest condition, i.e., the shallowest sweep.

### Thresholds at d′ = 0.77

**Figure 6:**
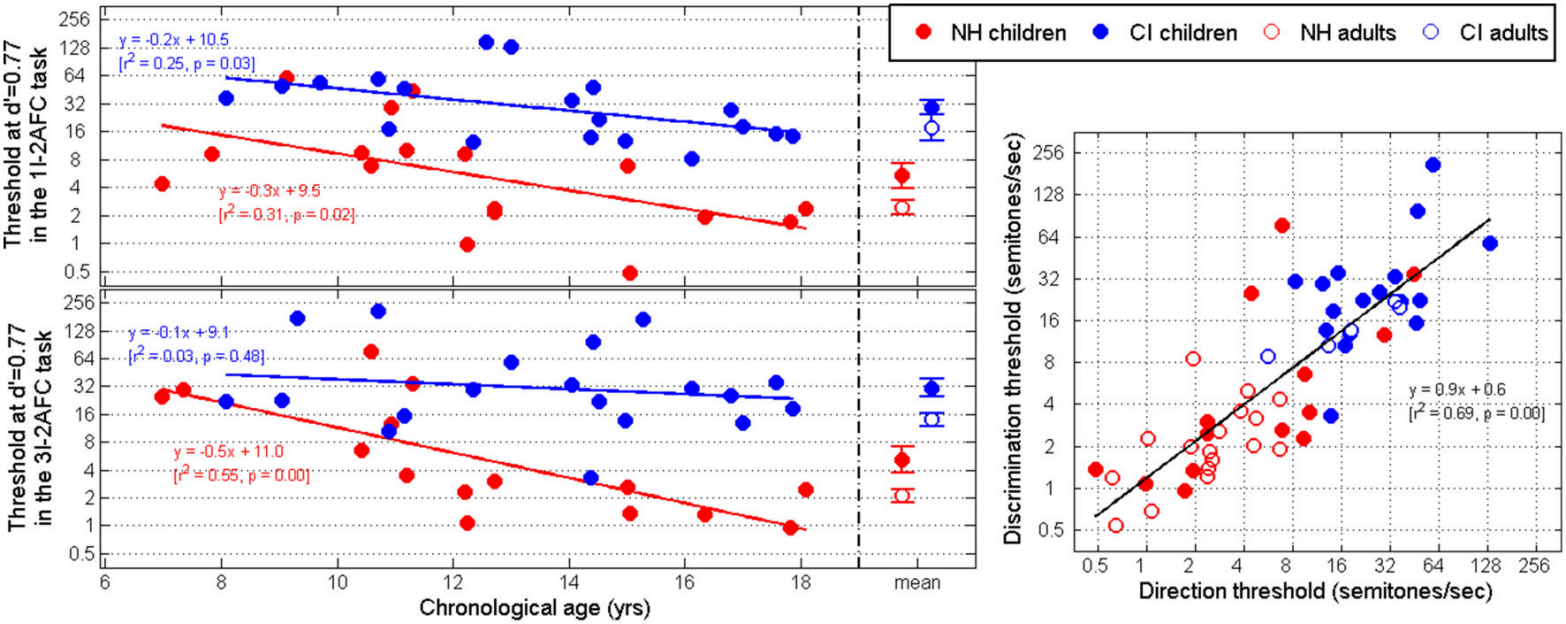
**Thresholds extracted at a d′ value of 0.77 for the direction (top-left) and discrimination (bottom-left) tasks, as a function of the child's chronological age**. Adult data are represented only as means. **(Right)** Correlation between thresholds in the two tasks, including individual adult subject data.

We now turn to individual subject data. Figure 6 shows the thresholds extracted from the Weibull fits at a d′ of 0.77 in the direction (top-left) and discrimination (bottom-left) tasks, as a function of the chronological age of each child. Only the mean thresholds are represented for adults. Individual data are plotted in the right panel in both tasks. Two NH children and three CI children produced near-chance data across all rates in the direction task. This was also the case for two CI children in the discrimination task (one who also failed the direction task). For those subjects, a threshold was not measurable at a d′ of 0.77, and they were absent from the figure, as well as further statistical analyses.

Thresholds were consistent between the two tasks, resulting in a strong correlation on the right panel. The average thresholds, represented on the top and bottom left panels, also lead to the same conclusions in both tasks. Namely, NH adults performed the best, with an average threshold of 2.5 and 2.1 semitones/sec (for each task, respectively). NH children performed worse, with an average threshold of 5.4 and 5.2 semitones/sec, and a larger variance. On the right panel, six of these NH children's thresholds were comparable with the NH adult thresholds, but eight other children had relatively poor performance. The CI populations obtained higher thresholds in comparison with the NH populations. CI adults had an average threshold of 17.7 and 14.1 semitones/sec, while CI children had an average threshold of 29.0 and 30.7 semitones/sec, respectively. As expected, variance was large for CI children as well. To address these results more formally, an analysis of variance was performed on the log-transform of thresholds, with two between-subjects factors: age (children vs. adults), and hearing status (NH vs. CI). In the direction task, there was a main effect of age [*F*_(1, 55)_ = 4.8, *p* = 0.033], and hearing status [*F*_(1, 55)_ = 39.3, *p* < 0.001], but no interaction [*F*_(1, 55)_ = 0.2, *p* = 0.626]. In the discrimination task, there was a main effect of age [*F*_(1, 53)_ = 7.0, *p* = 0.011], and hearing status [*F*_(1, 53)_ = 33.7, *p* < 0.001], but no interaction [*F*_(1, 53)_ < 0.1, *p* = 0.855]. The results of the statistical analysis were therefore consistent between the two tasks and confirmed (1) that CI listeners presented considerable deficits in their sensitivity to dynamic pitch relative to their NH peers, and (2) that adults had better sensitivity than children, in both populations.

In addition, there were significant correlations between thresholds and chronological age for NH children in both tasks, and this was also the case for CI children in the direction task only. The role of this developmental factor for the CI population is discussed next in relation to other factors.

### Experience-related factors

A multiple regression analysis was performed with (1) age at implantation, (2) age at profound hearing loss, and (3) duration of CI experience on the log-transform of the thresholds at d′ of 0.77 for CI children. The partial correlation relative to age at implantation was *r* = −0.295 (*p* = 0.055) and *r* = −0.125 (*p* = 0.505) in each task, respectively. The partial correlation relative to age at profound hearing loss was *r* = 0.389 (*p* = 0.259) and *r* = 0.652 (*p* = 0.094), and the partial correlation relative to duration of CI experience was *r* = −0.207 (*p* = 0.033) and *r* = −0.064 (*p* = 0.595). Thus, there was little evidence that age at implantation or age at profound hearing loss were relevant factors to dynamic pitch sensitivity. Duration of CI experience might perhaps play more of a role but, for this early-implanted population, it is difficult to know whether the effect is really driven by CI experience or simply chronological age as shown in the top panel of Figure 6. Note that chronological age is simply the sum of age at implantation and duration of CI experience. This co-linearity prevents a multiple regression analysis based on all four factors at once.

## General discussion

### Deficits in line with previous reports

The present study showed that CI children obtained much higher thresholds than their NH peers in their sensitivity to dynamic changes in pitch. These thresholds corresponded to sweeps that, within the 300-ms duration, covered 1.6 semitones and 9 semitones, respectively. Knowing that static thresholds were about 10–20 cents for NH children and 2–3 semitones for CI children (Deroche et al., 2014), the dynamic thresholds observed here represent F0 ranges that are several times larger than the static sensitivity. From inspection of the right panel in Figure 6, it is noteworthy that thresholds for the two populations, NH and CI, overlapped to a small extent. Four NH children had unexpectedly high thresholds and two CI subjects (one child and one adult) had thresholds between 8 and 16 semitones/sec, i.e., still in the vicinity of NH variability. This is quite a different pattern from our previous results on static pitch sensitivity (Deroche et al., 2014) where thresholds in the two populations did not overlap at all. The authors suggested that the envelope coding strategies inherent to current CI processing could pose a limit on the best pitch sensitivity that could be achieved by a CI user. Perhaps this conclusion should be moderated in the light of the present overlap. At least in a dynamic pitch task, and presumably more in linguistic tasks, there seems to be more room for an individual CI user to surpass typical expectations.

One goal of this study was to determine whether the deficits that resulted from CI processing were smaller or larger with adults than with children. The lack of interaction between age and hearing status in both tasks suggests that the deficits in dynamic pitch sensitivity are, on average, the same for children and adults. On the other hand, adults performed overall better than children, and older children performed better than younger ones. Therefore, there is certainly a clear role for chronological age but (based on present results) this is not accompanied by experience-related factors specific to CI users.

Perhaps most relevant to the present study, Luo et al. (2014) asked Mandarin-speaking CI children to discriminate between a high-level flat tone (Tone 1) and a mid-level rising tone (Tone 2). Using a continuum of linear F0 sweeps between Tone 1 and 2, they measured the progressive shift in response to Tone 2. Looking at the 29 and 71% points in their averaged psychometric functions (for CI alone and without context that could influence lexical tone normalization), we can derive comparable estimates of thresholds at a d′ of 0.77 which amount to +20.3 semitones/sec and -16.2 semitones/sec. This sensitivity fits nicely between the averaged thresholds for CI adults and CI children of this study, which seems reasonable given that the children used in their study were older (10–20 years with a mean of 15 years of age). Furthermore, age at implantation and duration of CI experience were not relevant factors in their study either.

The present study adds to the growing body of evidence indicating pitch-related deficits reported in the literature for this young and early-implanted population. CI children between 4 and 16 years old, listening in free-field conditions with their clinically assigned settings, displayed higher thresholds for pure tones discrimination at 0.5, 1, and 3 kHz, relative to their NH peers (Kopelovich et al., 2010). In other studies, CI children had difficulties perceiving and producing tonal contrasts in Cantonese (Barry et al., 2002; Ciocca et al., 2002) or in Mandarin (Peng et al., 2004). English-speaking CI children had difficulties differentiating between a question and a statement, both in terms of perception and in terms of production (Peng et al., 2008). CI children also display considerable deficits in recognizing emotion in voice (Most and Aviner, 2009; Ketelaar et al., 2012; Nakata et al., 2012; Volkova et al., 2013; Wang et al., 2013; Chatterjee et al., 2015). To summarize, it is clear that CI children suffer both from poor pitch sensitivity and difficulties with pitch-dominant cues in speech perception tasks, but the causality between the two remains to be substantiated, which was the goal of the subsequent section.

### Predictive power

We now turn to the predictive power of sweep thresholds for other tasks, specifically two recent studies related to pitch sensitivity. One was a standard discrimination task of static pitch sensitivity (Deroche et al., 2014). The other was a study on voice emotion recognition using a 5AFC task (Chatterjee et al., 2015). It was therefore of interest to correlate performance between the different tasks. This was possible because some of the subjects that participated in the present study had taken part in the previous studies. In addition, some subjects who had not taken part in the previous studies at the time, but did participate in this study were administered the previous experiments to increase the number of subjects common to the different tasks. We aimed to address two questions: (1) whether there is any fundamental difference between the sensitivity to static changes in pitch and the sensitivity to dynamic changes in pitch; and (2) whether basic psychoacoustic measures of pitch sensitivity could eventually be used in the clinic as a tool to quickly grasp an idea of a given subject's ability to use voice pitch information for speech-related purposes.

**Figure 7:**
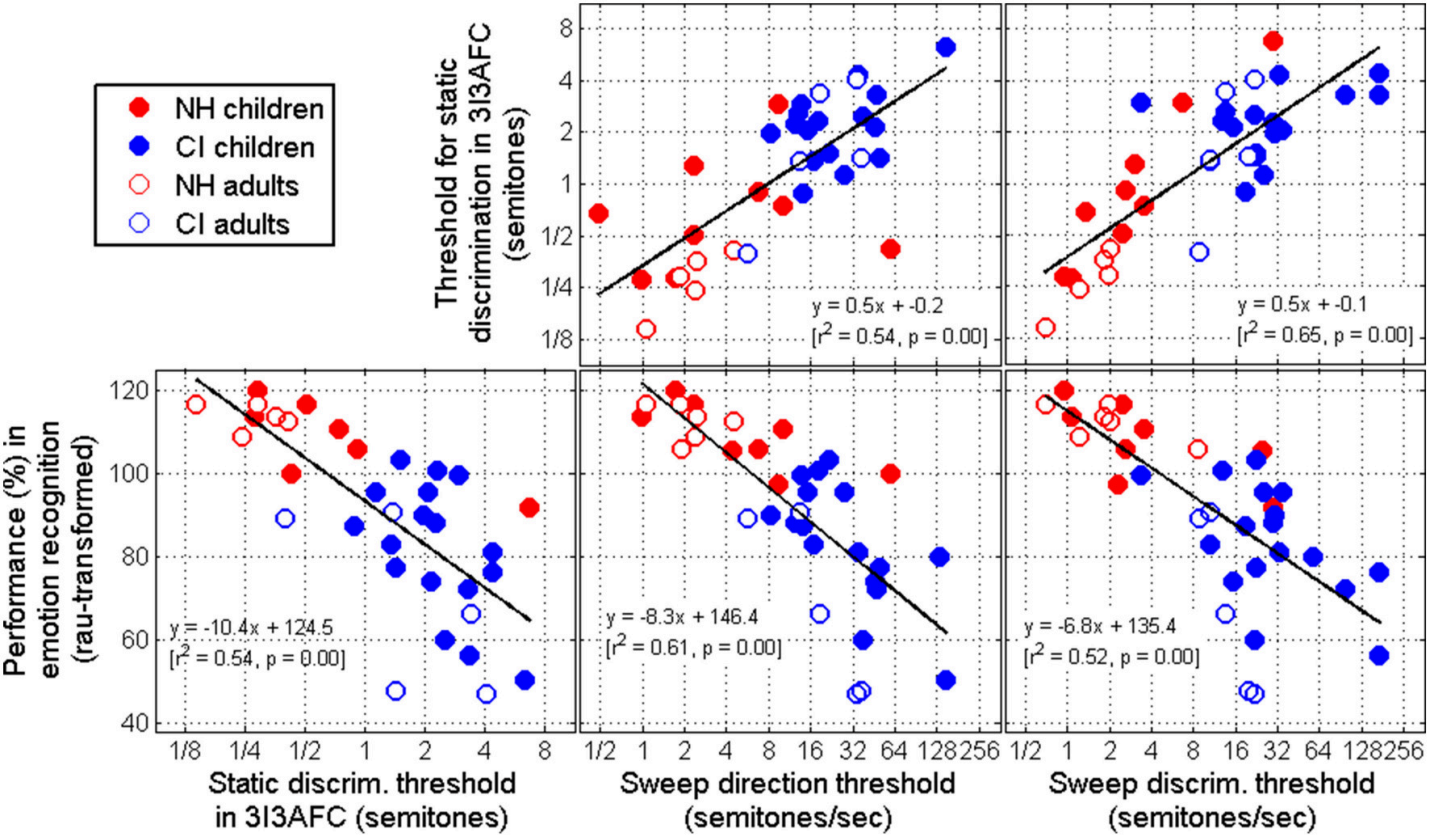
**(Top) Correlations between thresholds obtained in a 3I-3AFC task of static F0 discrimination with sweep thresholds**. **(Bottom)** Correlations between static and dynamic F0 thresholds with performance in a task of voice emotion recognition. All cases are significant, and sweep direction thresholds hold the most predictive power.

The two top panels of Figure 7 show that sensitivity to static changes in pitch was significantly correlated with sensitivity to dynamic changes in pitch. This result held true within the NH population alone, and within the CI population alone. This is perhaps not surprising but certainly reassuring: these tasks require fine coding of F0 information without which thresholds rise considerably. Besides, there was a stronger relationship between the dynamic discrimination task and the static discrimination task (top-right panel) than between the dynamic direction task and the static discrimination task (top-middle panel). This suggests that a direction task might engage some additional demands that are not required when listeners simply look for “the odd interval.”

The bottom panels of Figure 7 show that sensitivity to pitch—whether it was measured via a 3I3AFC static discrimination task, a 1I2AFC dynamic direction task, or a 3I2AFC dynamic discrimination task—was strongly correlated with performance by the same subjects in a voice emotion recognition task (subset of the data presented by Chatterjee et al., 2015). This result is quite important as it gets us closer to a causality relationship between pitch sensitivity and the use of prosody: the subjects who had the most difficulties in recognizing the speaker's communicative intent were these same subjects who had the poorest sensitivity to pitch. Note that two genders were used in the emotion recognition study, a female and a male voice. This analysis was performed for each gender separately, but the results were essentially the same, so performance was, here, averaged over the two genders for simplicity. Furthermore, these three correlations were also significant within the NH population alone and within the CI population alone. Note that the results of Luo et al. (2014) differ in this respect as CI children's perceptual boundaries were not correlated with their tone recognition scores. Perceptual boundaries were defined at 50% responses, corresponding to a d′ of 0, so we cannot infer whether, in their study, correlations would have occurred at higher d′ values.

The amount of variance explained by all three tasks is broadly similar, suggesting that any of these tasks could in principle provide useful indication of speech prosody recognition. However, given the anomalies observed in the sweep direction task, this particular measurement might be a risky choice. Surely, a task where there are the fewest concerns about measurement validity and response bias should be the focus of clinical tool development.

Finally, all these analyses were performed at other values of d′, simply to make sure that the choice of 0.77 did not lead to any particular case. Thresholds were re-extracted from the Weibull fits for the two sweep tasks as well as the static tasks, between d′ values of 0.05–2 in 0.05 steps, and the same analyses were replicated. Overall, the correlations aforementioned between psychoacoustic tasks were stable, i.e., observable, over a relatively broad range of d′ values, being strongest between 0.6 and 0.9. Toward high values of d′, beyond 1.5, the sweep direction thresholds were less reliable due to the influence of the anomalies discussed earlier, and became therefore less consistent with static thresholds and voice emotion recognition scores.

### Circumvent the anomalies of the detection task

In the eventuality that a researcher or clinician might be interested in using the sweep direction task in the future, one may want to optimize it somewhat. The question immediately arises as to whether the anomalies aforementioned could have been avoided. We chose to always use the same F0 range for up- and down-sweeps. This was clearly a necessity given that listeners appeared to rely on the F0 range even with shallow sweeps within the 100–150 Hz range (see Section F0 Roving of Results). Had the *starting F0* been fixed, the up-sweeps would have covered a high pitch range and the down-sweeps would have covered a low pitch range, meaning that performance would have improved for the wrong reason and d′ would have been massively overestimated. So it was certainly essential to have up- and down-sweeps cover the exact same range and this represents a tight constraint. Increasing the F0 roving (e.g., a 3-octave range from 100 to 800 Hz) would also have failed to prevent the problem. Of course, this would have let many of the shallow sweeps fall in a high pitch range, but a steep sweep based on F0 close to 800 Hz would have obviously gone well beyond 800 Hz, pushing the problem higher up. Alternatively, one may choose to have the median F0 (not the base F0) roved between 100 and 150 Hz, such that steep sweeps would not elicit a higher or lower pitch range from a long-term average than shallow sweeps. But such steep sweeps would still elicit a very broad F0 range and it is not impossible that listeners would still show a bias in favor of one direction over the other simply based on the width of the pitch range elicited. Besides, stimuli sweeping six octaves with a median F0 of 100–150 Hz would engage very low F0s for half of the stimuli duration which may no longer be relevant to the human voice range. It is also interesting that CI users showed the same biases as NH listeners, despite having much poorer F0 resolution. Somehow, they too must construct trial after trial an internal reference of pitch range where most sweeps are expected to vary. Even though this internal reference might be coarser, it produced the same response biases. As a conclusion, it is not clear to us how such anomalies could be prevented. Extracting thresholds at a d′ of 0.77 was actually fortuitous in the present study, as by doing so, the analyses largely missed the influence of these anomalies.

Perhaps, the best avenue for future endeavors is to optimize the stimulus duration. With longer stimuli, listeners would have more glimpses at different time windows to perceive the sweep direction. This might therefore discourage a strategy based on long-term average of F0. For the small number children who had the most difficulties in performing these tasks (with 300-ms stimuli), we did in fact try longer durations (not shown here) and performance improved. Perhaps more interesting is that performance improved with longer stimuli even when adjusting the sweep rate so as to cover the same F0 range, reflecting the benefit of making more modulation cycles available. Luo et al. (2010) showed exactly these effects with direct electrical stimulation on CI adults: they measured discrimination thresholds for amplitude modulation rate for 50-, 100-, and 200-Hz baselines with durations of 50, 100, 200, and 400 ms. For the 50-Hz baseline, and to a smaller extent for the 100-Hz baseline, sensitivity improved with duration up to 100 ms, suggesting that subjects may need 5–10 modulation cycles to accurately process changes in modulation rate. These results are also consistent with earlier reports on pulse train rate discrimination by CI users (Tong et al., 1982) and NH subjects (Plack and Carlyon, 1995). In the present study, the duration of 300 ms would have offered more than 10 cycles for any harmonic complex beyond 33-Hz F0. Thus, it may actually be surprising that children would have shown benefits of durations longer than 300 ms. A formal investigation of duration effects for dynamic pitch sensitivity would cast a very informative light on the temporal integration of CI children and hopefully lead to an optimized stimulus duration for the direction task. However, one should bear in mind that the choice of 300 ms is at least relevant for pitch processing at the syllabic rate; for tasks such as voice emotion recognition, much longer time scales (e.g., 3 s) would have to be considered.

## Summary

Sensitivity to F0 sweeps was measured in a 1I-2AFC and in a 3I-2AFC constant-stimulus procedure with a child-friendly interface. Psychometric functions were measured in four groups of listeners (children and adults, with and without cochlear implants), for up- and down-sweeps at logarithmically spaced rates. Subjects wearing cochlear implants were always tested on a single side, implanted first, and used their clinically assigned settings with envelope-based coding strategies. On one hand, implanted children and adults showed considerable deficits compared with their normally-hearing peers. On the other hand, children performed worse than adults, and this developmental factor was further corroborated by an effect of chronological age among normally-hearing children as well as implanted children in the direction task. There was however little influence of experience-related factors such as age at implantation, age at profound hearing loss, and duration of CI experience. Some anomalies occurred in the direction task, which sometimes resulted in non-monotonic psychometric functions for down-sweeps. They were presumably due to the fact that in the presence of very steep sweeps, some listeners disregarded the direction of the sweeps completely, and instead used the long-term average F0 as an alternative cue when it exceeded the F0 base roving range of 100–150 Hz. This problem is not easily circumvented but might benefit from durations longer than 300 ms and perhaps an explicit intervention by the experimenter not to follow such a strategy. However, the extraction of thresholds at a d′ value of 0.77 attenuated the influence of these anomalies and consequently, the present results were strongly correlated in between the two tasks. Thresholds for dynamic pitch changes were also correlated with thresholds for static pitch changes, and all correlated with performance in voice emotion recognition. A relatively quick measure of pitch sensitivity, be it static or dynamic, could therefore be used as a clinical tool to assess potential deficits that an implanted child may have in terms of reception of speech prosody information.

## Author contributions

MD designed the experiments, collected data (at the Johns Hopkins site), analyzed the data, and wrote the manuscript. AK and JC were responsible for data collection at the BTNRH site. CL was responsible for supervision of the project at the Johns Hopkins site, participated in conceptualization of the project, and contributed to manuscript writing. MC led the development of the rationale and conceptualization of the project, worked with MD on experimental design and contributed to manuscript writing.

### Conflict of interest statement

The authors declare that the research was conducted in the absence of any commercial or financial relationships that could be construed as a potential conflict of interest.
